# Cystatin C Is Downregulated in Prostate Cancer and Modulates Invasion of Prostate Cancer Cells *via* MAPK/Erk and Androgen Receptor Pathways

**DOI:** 10.1371/journal.pone.0007953

**Published:** 2009-11-23

**Authors:** Barbara Wegiel, Thomas Jiborn, Magnus Abrahamson, Leszek Helczynski, Leo Otterbein, Jenny Liao Persson, Anders Bjartell

**Affiliations:** 1 Department of Clinical Sciences, Division of Urological Cancers, Clinical Research Center, University Hospital Malmö, Lund University, Malmö, Sweden; 2 Department of Pathology, Clinical Research Center, University Hospital Malmö, Lund University, Malmö, Sweden; 3 Department of Laboratory Medicine, Clinical Research Center, University Hospital Malmö, Lund University, Malmö, Sweden; 4 Department of Laboratory Medicine, Division of Clinical Chemistry and Pharmacology, Lund University Hospital, Lund University, Lund, Sweden; 5 Department of Surgery, Beth Israel Deaconess Medical Center, Harvard Medical School, Boston, Massachusetts, United States of America; Baylor College of Medicine, United States of America

## Abstract

Cystatin C is believed to prevent tumor progression by inhibiting the activities of a family of lysosomal cysteine proteases. However, little is known about the precise mechanism of cystatin C function in prostate cancer. In the present study, we examined the expression of cystatin C and its association with matrix metalloproteinases 2 (MMP2) and androgen receptor (AR) in a tissue microarray comparing benign and malignant specimens from 448 patients who underwent radical prostatectomy for localized prostate cancer. Cystatin C expression was significantly lower in cancer specimens than in benign tissues (p<0.001) and there was a statistically significant inverse correlation between expression of cystatin C and MMP2 (r_s_^2^ = −0.056, p = 0.05). There was a clear trend that patients with decreased level of cystatin C had lower overall survival. Targeted inhibition of cystatin C using specific siRNA resulted in an increased invasiveness of PC3 cells, whereas induction of cystatin C overexpression greatly reduced invasion rate of PC3 in vitro. The effect of cystatin C on modulating the PC3 cell invasion was provoked by Erk2 inhibitor that specifically inhibited MAPK/Erk2 activity. This suggests that cystatin C may mediate tumor cell invasion by modulating the activity of MAPK/Erk cascades. Consistent with our immunohistochemical findings that patients with low expression of cystatin C and high expression of androgen receptor (AR) tend to have worse overall survival than patients with high expression of cystatin C and high AR expression, induced overexpression of AR in PC3 cells expressing cystatin C siRNA greatly enhanced the invasiveness of PC3 cells. This suggests that there may be a crosstalk between cystatin C and AR-mediated pathways. Our study uncovers a novel role for cystatin C and its associated cellular pathways in prostate cancer invasion and metastasis.

## Introduction

Prostate cancer (PCa) remains the most common and second most lethal tumor in males in the Western World [1]. Approximately one-third of treated patients will relapse and no curative treatment currently exists for metastatic disease [2]. The progression through hormone-dependent to castration resistant and metastatic prostate cancer is poorly understood. The processes of invasion and metastasis by tumor cells are dependent on their ability to degrade surrounding proteins and other tissue components. The proteolytic enzymes and proteases such as collagenase and cathepsins are necessary for this purpose, and thus play crucial roles in multiple steps of cancer growth and metastasis [3], [4]. Among proteases, the matrix metalloproteinases MMPs and lysosomal cathepsins B have been attributed major roles in prostate cancer progression [5]–[9] [10], [11]. Recently, MMP2 was also linked to an invasive phenotype of prostate cancer cells [12] and expression of MMP2 in malignant prostatic epithelium was demonstrated to be an independent predictor of prostate cancer disease-free survival [13].

Cystatin C is a secreted cysteine protease inhibitor that regulates bone resorption, neutrophil chemotaxis, and tissue inflammation as well as resistance to bacterial and viral infections. It also serves as a potent inhibitor of cathepsin B and other human lysosomal cysteine proteases [14]. Cystatin C is also known to be a better marker for renal injury than creatinine [15], [16]. By inactivating cathepsin protease activity, cystatin C inhibits cancer cell invasion and metastasis [17], [18]. Abnormal serum levels of cystatin C or cathepsin B/cystatin C complex have been suggested as diagnostics and prognostic indicators for cancers of skin, colon and lung [19]. Cystatin C has been suggested to play an important role in neuroendrocrine differentiation of prostate cancer [20]. More recently, serum cystatin C has been proposed as useful marker of increased osteoblastic activity associated to bisphosphonate treatments in prostate cancer patients with bone metastasis [21]. However, the role of cystatin C in prostate cancer progression and its associated cellular and molecular networks remain to be investigated.

Recent studies have shown that during tumorigenesis and metastasis, various proteolytic cascades consisting of enzymes such as cysteine proteases and MMPs act in a synchronized manner and aid in tumor growth, invasion into surrounding tissues [10]. Cathepsin B has been implicated in the degradation of the extracellular matrix (ECM) either in secreted form in the extracellular space or attached to the cell surface [10]. In particular, MMP-2 and MMP-9 have been suggested to be associated with prostate cancer metastasis, as high levels of these proteins were measured in plasma and urine in patients with metastatic disease [5], [22], [23]. MMP9 has also been studied intensively and is though to play a major role in two important aspects of tumor progression, angiogenesis and vasculogenesis [8].

The metastatic process involves the coordination of several cellular and signal-transduction pathways that allow cancer cells to proliferate, remodel their surrounding environment, invade to distant site and form new tumors. MAPK signalling pathways play an important role in inducing secretion of proteolytic enzymes that degrade the basement membrane, enhancing cell migration and maintaining tumor cell growth [7]. Increases in MAPK activity have been observed in advanced PCa suggesting that a constitutively active Ras pathway might be associated with prostate cancer progression and metastasis [7], [24]. Importantly, MAPK activation is linked to development of androgen-independent prostate cancer, now commonly termed castration-resistant prostate cancer (CRPC) [25], [26], [27].

Androgen receptor (AR), a member of the superfamily of ligand-activated nuclear receptors, plays a central role in the pathogenesis of primary and metastatic prostate cancer [28], [29]. AR gene amplification is found in one third of advanced prostate cancers and is believed to contribute to progression and metastasis of prostate cancer [30], [31], [32], [33]. AR does not act independently in the regulation of tumor growth but requires interaction with co-regulators [34]. Mutations in the gene or message of the AR are the reasons for the increased androgen sensitivity of those tumors. Local autocrine production of dihydrotestosterone and testosterone in prostate cancer cells diminishes the castration effect [35]. The crosstalk between AR and other signaling pathways (e.g. MAPK) as well as changes in AR co-regulators [34] accelerate the ligand independent activation of AR. Thus, AR plays a vital role in both clinically localized and advanced prostate cancers.

The present study aimed to evaluate the expression of cystatin C and its clinical relevance in prostate cancer, and to elucidate a novel role for cystatin C in prostate cancer invasion. Cystatin C is associated with the proteolytic cascade and MAPK/Erk pathway together with AR may act in a synchronized manner to promote tumor growth and invasion into surrounding tissues. Here, we established for the first time a functional link between cystatin C and MAPK-Erk signalling and AR-mediated pathways in prostate cancer cells.

## Results

### Tissue Expression of Cystatin C in Prostate Cancer Is Associated with MMP2 as a Marker for Invasiveness and Clinical Outcome

**Figure 1:**
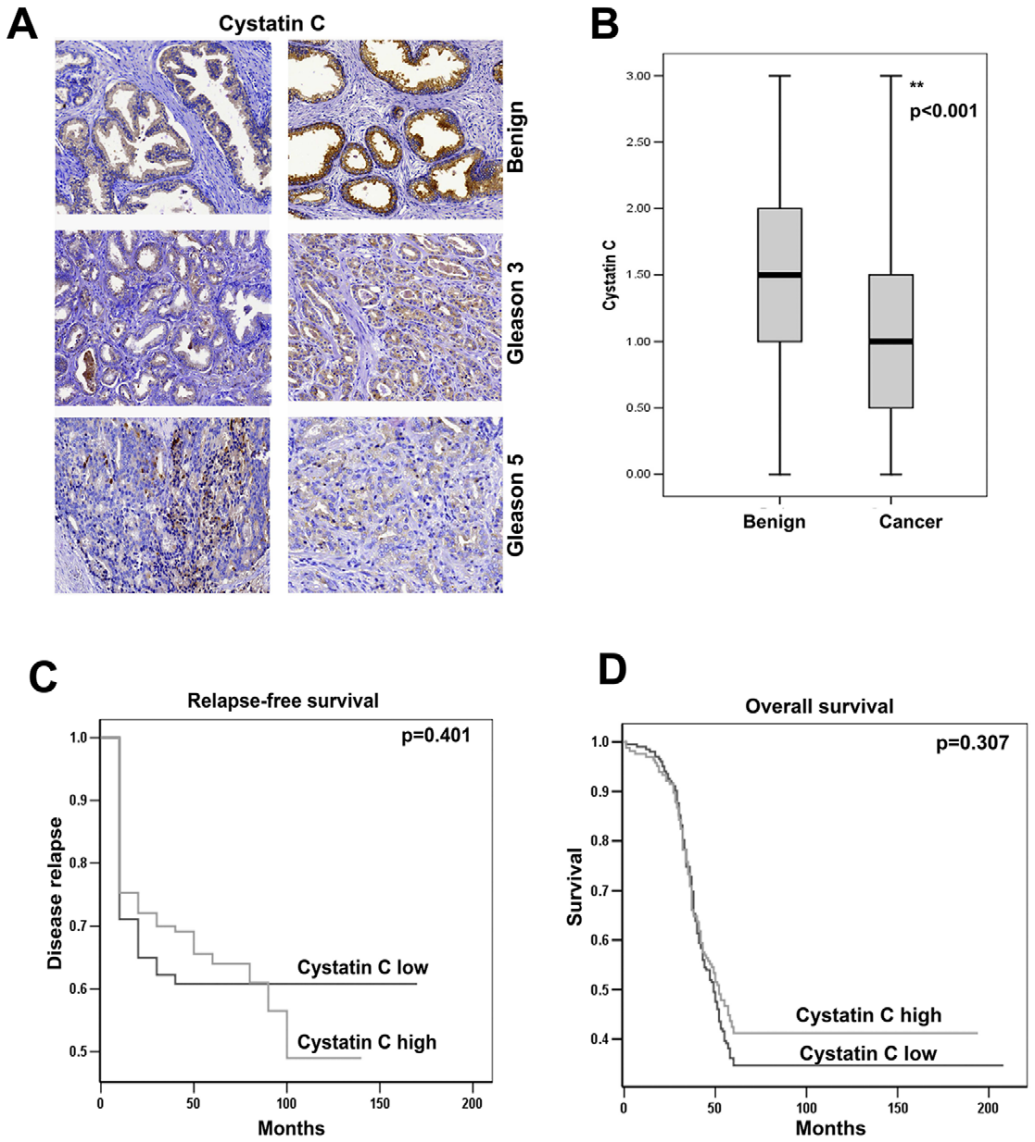
Immunohistochemical analysis of cystatin C expression in TMA with malignant and benign samples from patients with localized prostate cancer. **A**). Immunohistochemical analysis of cystatin C expression in benign and PCa specimens. Sections representing benign, tissue and tumors of Gleason grade 3 and grade 5 are shown. Representative pictures were obtained using a 40x objective. **B**). The graph of quantitative analysis of immunohistochemical staining of Cystatin C (score 0-negative, 1- moderate, 2- strong, 3- very strong) shows the comparison between 448 benign and cancer specimens (average staining of duplicates of each specimen). The paired Wilcoxon's rank sum test analyses were used to assess the comparison between the groups. The mean values of intensities of staining (horizontal lines) with error bars representing 95% confidence intervals for the mean are shown. The boxes represent the distribution of the expression of cystatin C in the groups. **C**) Overall survival in patients with high or low expression of cystatin C in prostate cancer samples. Kaplan-Meier survival analysis was performed. **D**) Survival curves of time to relapse as evaluated as a biochemical recurrence measured as by raise of PSA [57] for the low (intensity score 0–1.5) and high (intensity score 2–3) cystatin C expression.

Decreased cystatin C mRNA expression has been reported in several types of solid tumors including breast cancer, colon cancer and renal carcinoma [36], [37]. However, the specific role of cystatin C protein expression in prostate cancer progression and its association with clinical characteristics has not been reported. We utilized a tissue-microarray (TMA) containing specimens from benign prostatic tissue and malignant tumors from 448 patients who underwent radical prostatectomy for localized prostate cancer. Expression of cystatin C was examined by immunohistochemistry. Virtually all benign specimens showed markedly high cytoplasmic protein expression of cystatin C while the matched prostate cancer tissues consistently displayed weaker or undetectable immunostaining (**Figure 1A, B**). The difference was statistically significant (p<0.001), suggesting that cystatin C protein expression is, in general, down-regulated in prostate cancer compared to benign epithelium. We then subdivided tumor samples into two groups based on Gleason grades of individual core biopsies, Gleason grade 2 or 3 (Group 1) vs. Gleason grade 4 or 5 (Group 2) [38]. We found decreased expression of cystatin C in 61% tumor samples from group 1 compared with 72% of the samples in Group 2 (**Figure 1A**). Both MMP2 and MMP-9, in particular, have been found to be associated with prostate cancer metastasis [13], [39]. Recently, cystatin C was identified as a novel substrate for MMP2 in cell-based proteomic analysis, suggesting that there is a direct functional link between MMP2 and cystatin C [40]. We therefore explored a possible association between cystatin C expression and the expression of MMP2 in the patient samples by immunohistochemical analysis of our tissue microarray construct (TMA). Interestingly, expression of cystatin C protein was inversely associated with MMP2 in the 448 patient material which was statistically significant (r_s_^2^ = −0.056, p = 0.05). We further investigated whether cystatin C expression correlated with clinical outcome in prostate cancer patients, and we divided patients into two groups based on the level of cystatin C expression: cystatin C high (intensity of staining 2 or 3) or cystatin C low (intensity of staining less than 2) group. We found no significant difference in clinical parameters including Gleason score, T stage/Pathology stage, metastasis free time, pretreatment PSA levels, Biochemical recurrence PSA levels, Biochemical free time and positive surgical margins between cystatin C high and cystatin C low group (**Table 1**). We then investigated whether clinical outcome including overall-survival (OS) differed between cystatin C high and cystatin C low patients. Patients in the cystatin C high group at 100 months from diagnosis had an OS of approximately 40% compared to 25% for patients in the cystatin C low group (p = 0.307) (**Figure 1C**). Although we did not achieve statistically significance, there is a clear trend that patients with low level of cystatin C expression had worse outcome compared with those with higher levels. When we assessed whether biochemical recurrence-free survival differed between the groups, there was also no significant difference (p = 0.401) (**Figure 1D**).

**Table 1:** Demographic and clinical data for PCa patients with high or low expression of cystatin C.

N = 435, Mean values	Low Cystatin C (staining under 2) (n = 315)	High Cystatin C (staining 2–3) (n = 120)	P values
Gleason score	6.2	6.3	0.139
Clinical T stage	1.56	1.64	0.169
Pathological T stage	2.48	2.56	0.135
Metastasis free time	2.32	2.75	0.806
Survival time (months)	268	264	0.479
Progression free time/survival	29.4/61.3	30.3/64.17	0.793
Pretreatment PSA	9.0	8.78	0.743
BCR PSA	0.695	0.463	0.266
BCR free time	30.22	32.38	0.507
Positive surgical margin	0.54	0.56	0.635

BCR: Biochemical Recurrence.

### Tissue Expression of Androgen Receptor Is Inversely Associated with Cystatin C Expression

**Figure 2:**
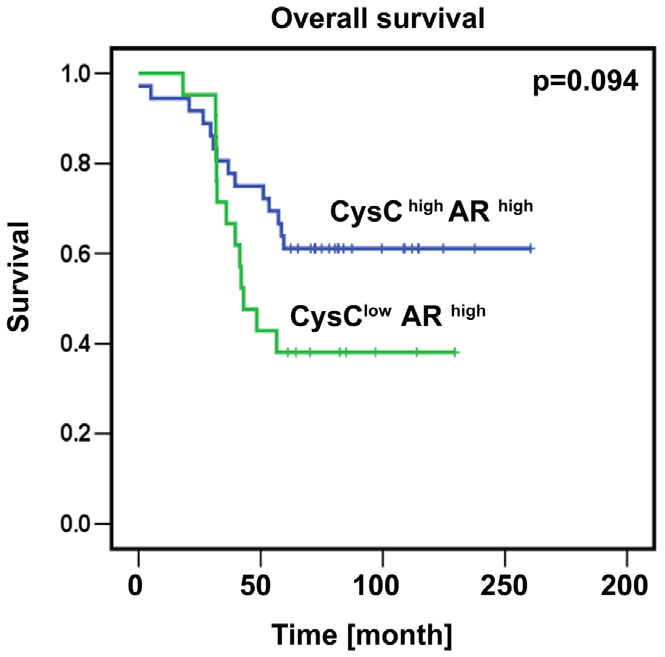
Kaplan-Meier survival analysis in 99 patients with advanced prostate cancer. Overall survival in a group of 99 patients with the most advanced prostate cancer (Gleason grade 4–5) which were characterized by high expression of AR and were separated to different groups based on cystatin C levels (low- intensity score 0–1.5 and high- intensity score 2–3).

Amplification and mutations of AR gene are identified as critical factors related to the poor prognosis of prostate cancer. We wanted to investigate whether cystatin C expression in combination with AR may predict outcome of the disease. We evaluated expression of cystatin C in a subset of our TMA samples comprising 99 patients with the most advanced disease and had high level of AR expression. We divided these 99 patients into two groups: the cystatin C-low/AR-high group and cystatin C-high/AR-high group (**Figure 2**). Patients with low cystatin C levels and high AR expression had lower overall survival (40%, at 100 months) compared with patients with high cystatin C levels and high AR expression (60%, at 100 months), although these results did not achieve statistical significance (p = 0.094). This indicates that patients who had low cystatin C and high AR expression tend to have worse outcome compared with patients with high cystatin C and high AR expression.

### Cystatin C Expression in Prostate Cancer Cell Lines

**Figure 3:**
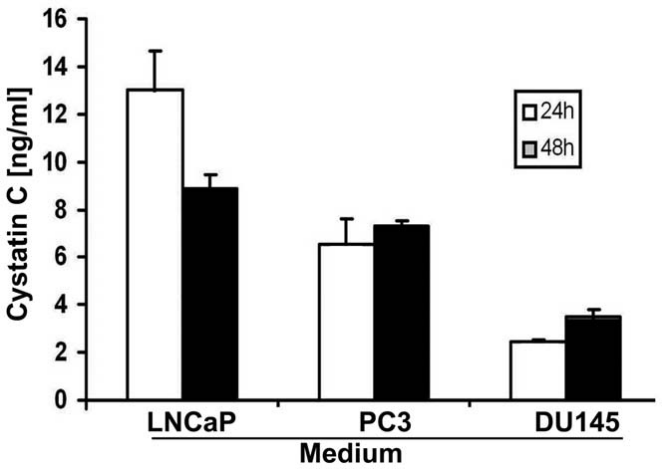
Cystatin C is expressed in the media of prostatic cells lines. ELISA assay of supernatants from three different prostate cancer cells lines, which were plated 24 h or 48 h before experiment was carried out. Data are shown as average of triplicates±SD for 3 experiments.

Because cystatin C was down-regulated in prostate cancer tissues and associated with increased expression of MMP2 that is known to contribute to tumor invasion and metastasis, we wanted to investigate the role of cystatin C expression in prostate cancer cell growth, survival and invasion. We examined cystatin C expression in three prostate cancer cell lines including the androgen-sensitive LNCaP cells and androgen-insensitive PC3 and DU-145 cells (**Figure 3**). Cystatin C protein concentrations in the supernatants were measured by ELISA, after culturing the cells in serum-free media for 24 and 48 hours, respectively. Interestingly, LNCaP cells which are known to be non-invasive produced high levels of cystatin C, while the invasive cell lines PC3 and DU-145 showed lower levels of cystatin C secretion (**Figure 3**). PC3 cells, with a moderate expression level of cystatin C were chosen for subsequent functional studies to evaluate the effects of forced overexpression and knockdown of cystatin C on PC3 cell invasion.

### Down-Regulation of Cystatin C Is Linked to Invasion of PC3 Cells

**Figure 4:**
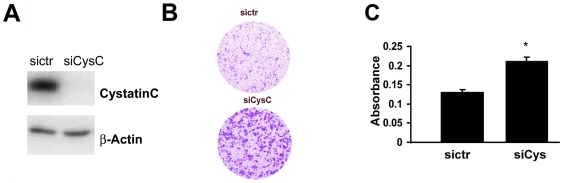
The effect of knockdown of cystatin C on the invasion of PC3 cells. **A**). Immunoblotting of cystatin C in PC3 cells after treatment with control (siCtr) and cystatin C (siCys) siRNA. **B–C**) Invasion assay in matrigel- coated Boyden chambers of PC3 cells with knockdown of cystatin C. Representative image of invading cells is shown in (**B**) and quantitative analysis of invasion by measuring absorbance after staining of invading cells with Cell Stain Solution containing crystal violet supplied in the Transwell Invasion assay (Chemicon, Millipore, CA) (**C**) Data±SD are representative for at least 3 experiments;* p<0.01 (Student T-test).

Since PC3 cells are invasive, express moderate level of cystatin C and have lack functional AR, thus can be an excellent model for combined studies on cystatin C and AR function. PC3 cells were transfected with cystatin C siRNA vector or control siRNA vector for 24 hours. Immunoblot analysis confirmed that cystatin C siRNA specifically blocked the protein expression of cystatin C in PC3 cells (**Figure 4A**). To examine the effect of cystatin C knock down on modulating the invasive activity of PC3 cells, in vitro invasive activity assays were performed to assess the proportion of PC3 cells expressing cystatin C siRNA or control siRNA that have invaded through matrigel coated membranes. A significantly higher proportion of PC3 cells, transiently transfected with cystatin C siRNA, migrated through matrigel coated chambers compared to that of PC3 cells transfected with control siRNA (**Figure 4B–C**). Next, we assessed whether cystatin C knockdown might have effect on proliferation of prostate cancer cells. PC3 cells transfected with cystatin C siRNA or control siRNA were subjected to BrdU incorporation assay. Cellular proliferation was assessed after 24 hours or 48 hours of transfection. No significant differences in proliferation rates between PC3 cells transfected with siRNA to cystatin C or control siRNA were observed (data not shown), suggesting that inhibition of cystatin C had no effect on PC3 cell proliferation. We also evaluate the effect of cystatin C on cell cycle distribution. Flow cytometric analysis was performed in cells transfected with cystatin C siRNA or control siRNA, we did not observe that silencing of cystatin C had any significant influence on the distribution of cell cycle phases in PC3 cells (data not shown). Next, we wanted to test whether inhibition of cystatin C may have any effect on the survivals of PC3 cells in response to the treatment with cytotoxic agent. PC3 cells transfected with siRNA to cystatin C or control siRNA were treated with the cytotoxic agent camptothecin at 10 ng/ml to induce apoptosis. Treatment of PC3 cells with camptothecin successfully induced cell death in PC3 cells. PC3 cells transfected with siRNA against cystatin C, however, showed no significant difference in camptothecin-induced apoptosis as compared with PC3 cells transfected with control siRNA (data not shown).

**Figure 5:**
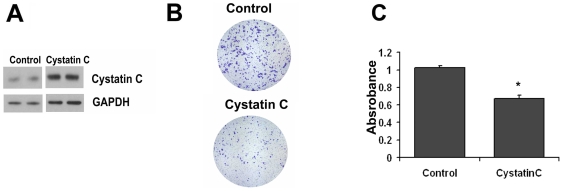
Cystatin C decreases invasion of prostate cancer cells. **A**) Immunoblot of cystatin C in PC3 cells after stable transfection with pcDNA3.1 and cystatin C-pcDNA3.1 plasmids. Stable clones were established after 2 weeks of selection on neomycin. **B–C**) Invasion assay of PC3 cells with overexpression of control or cystatin C plasmids. Representative images of cells are shown in **B** and quantification (absorbance) of data +SD from 3 independent experiments is shown in C. *p<0.05 (Student T-test).

Because inhibition of cystatin C in PC3 cells had significant effect on PC3 cell invasion, we therefore assessed whether overexpression of cystatin C might have inhibitory effects on the invasive behavior of PC3 cells. To induce overexpression of cystatin C in PC3 cells, PC3 cells were transfected with a cystatin C expression vector or with an empty expression vector. The stable overexpression of cystatin C in PC3 cells was achieved after antibiotic selection. Overexpression of cystatin C in PC3 cells was confirmed by immunoblot analysis (**Figure 5A**). When we investigated the effect of overexpression of cystatin C on PC3 cell invasion, we noted that a significantly lower proportion of PC3 cells overexpressing cystatin C migrated through Matrigel-coated Boyden chambers compared with cells expressing control vector (**Figure 5B–C**). Taken together, our current data suggests an important role for cystatin C to inhibit prostate cancer cell invasion.

### The Role of Erk2 Pathway in Cystatin C Mediated Effects on Invasion

**Figure 6:**
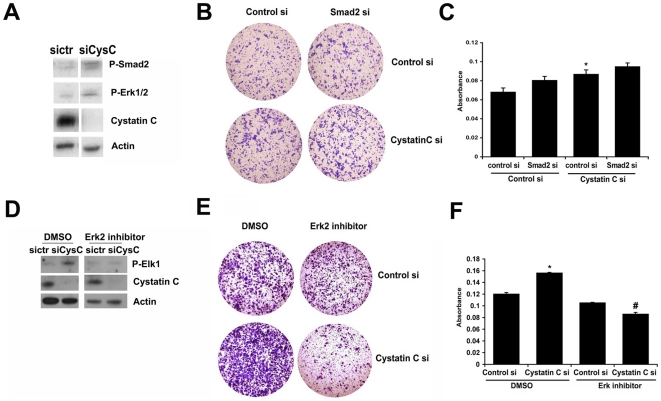
The role of Erk1/2 and Smad2 in cystatin C regulated invasion of PC-3 cells. **A**). Immunoblot analysis of P-Smad2 in PC-3 cells after silencing of Smad2. **B–C**). Invasion assay in PC-3 cells after silencing of Smad2 simultaneously with knockdown of cystatin C. The representative pictures are shown in **B** and quantitative results of 3 independent experiments are presented in **C**. *p<0.05 (Student T-test). **D**). Immunoblot with antibody against phosphorylated (Ser383)-Elk1 and cystatin C in the lysates from PC3 cells transfected transiently with siRNA cystatin C and control siRNA and co-treated with Erk2 inhibitor (25 µM). Note that Erk2 inhibitor blocks downstream phosphorylation of Elk1 a target of Erk2 in cells with knockdown of cystatin C. Data are representative for 2 experiments. **E–F**). Invasion assay in PC-3 cells after concomitant silencing of cystatin C and inhibition of Erk2 with selective inhibitor. The representative pictures are shown in D and quantitative results of 2 independent experiments are presented in **E**. *p<0.05, #p<0.01 (Student T-test).

Next, we wanted to investigate the cellular mechanisms and pathways by which cystatin C exerts its effect on PC3 cell invasion. MAPK signalling pathways and TGFβ pathways have been shown to have functional link with proteolytic enzymes including cathepsin family of proteins to promote the degradation of the basement membrane, enhances cell invasion and maintains tumor cell growth. Smad 2/3 proteins are the downstream effectors of TGFβ signaling and the target of MAPK/ERK pathways as well, we therefore wanted to investigate whether cystatin C may have a direct functional link to these signalling pathways. We firstly examined whether cystatin C may mediate the activities of MAPK and TGFβ pathways by regulating the expression and phosphorylation of Smad2 and Erk1/2 in PC3 cells. We examined phosphorylation of Smad2 and Erk1/2 in PC3 cells transfected with cystatin C siRNA or control siRNA (**Figure 6A**). Immunoblot analysis revealed that an increase in the level of phosphorylated Smad2 and Erk1/2 was observed in PC3 cells transfected with cystatin C siRNA, suggesting that both Smad2 and Erk1/2 may be the downstream effectors inhibited by cystatin C (**Figure 6A**).

To functionally examine the role of Smad2 activation in PC3 cells, we tested if targeted Smad2 knockdown had any effect on tumor cell invasion. We designed siRNA to specifically target Smad2. Inhibition of Smad2 phosphorylation via Smad2 siRNA mediated knockdown was achieved in PC3 cells and was confirmed by immunoblot (data not shown). In vitro invasive activity assays revealed that the proportion of migrating and invading PC3 cells were similar in PC3 cells transfected with Smad2 siRNA and in PC3 cells transfected with control siRNA (**Figure 6B–C**). We then performed simultaneously targeted knockdown of Smad2 and cystatin C in PC3 cells. PC3 cells that were co-transfected with cystatin C siRNA and Smad 2 siRNA or control siRNA were further applied on the invasion chamber for the invasion assay. There was no differences in the invasion rate between PC3 cells co-expressing cystatin C siRNA and Smad 2 siRNA and PC3 cells co-expressing cystatin C siRNA and control siRNA (**Figure 6B–C**), suggesting that inhibition of Smad2 had no additional effects on modulating invasion of PC3 cells expressing cystatin C siRNA and that Smad2 may be not involved in cystatin C-mediated tumor cell invasion.

Because an increase in the level of phosphorylated Erk1/2 was also observed in PC3 cells transfected with cystatin C siRNA in the above experiments, we therefore further investigated if activation of Erk1/2 mediated the inhibitory effect of cystatin C on PC3 cell invasion. Treatment with a MEK inhibitor (PD98059), a general inhibitor of MAPK pathways had no effect on the invasive behaviour of PC3 cells expressing cystatin C siRNA or control siRNA (data not shown). We decided to selectively inhibit the activities of Erk 1 or Erk2.

Firstly, we inhibited Erk 1 expression via siRNA targeted knockdown. Inhibition of Erk 1 had no effect on the invasion of PC3 cells expressing cystatin C siRNA (data not shown), suggesting that Erk1 may be not involved in cystatin C associated tumor cell invasion. Next, we used selective inhibitor of Erk2 activity, which blocked phosphorylation of Elk-1, a downstream target of Erk2, in PC3 cells expressing cystatin C siRNA (**Figure 6D**). We observed that Erk 2 inhibitor significantly inhibited the rate of invasiveness in cells expressing cystatin C siRNA compared to cells expressing control siRNA (**Figure 6E–F**). The Erk2 inhibitor had no effect on cell proliferation in PC3 cells under the same condition (data not shown). These results suggest that Erk2 may be a downstream target of cystatin C. This suggests that PC3 cells that have low cystatin C and high level of Erk2 activity may become more invasive compared with cells with normal level of cystatin C and Erk 2 activity. Further, cystatin C may mediate tumor cell invasion through MAPK/Erk2 signalling pathways.

### AR Enhances Cell Invasion in the Absence of Cystatin C

**Figure 7:**
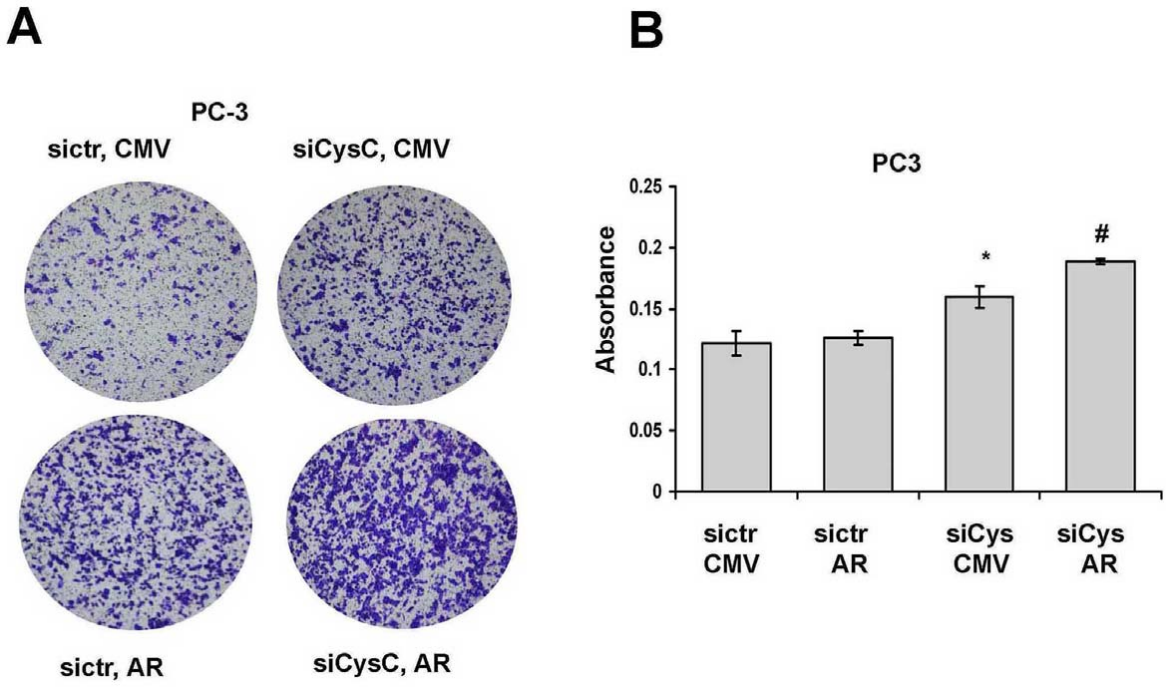
AR regulates the invasion in the absence of cystatin C. **A–B**). Invasion assay of PC3 cells transfected with control siRNA or against cystatin C and coexpressing AR. The pictures from representative experiment are shown in **A** and the quantitative absorbances of invading cells (*p<0.05, cystatin C siRNA versus siRNA control, #p<0.05 AR versus CMV, Student T-test) after stating with Cell Stain Solution supplied in the Transwell Invasion assay (Chemicon, Millipore, CA) are shown in **B**. The data are representative for 3 independent experiments.

AR is an important downstream target of MAPK/Erk pathways, and inhibition of Erk1/2 activity will lead to a decreased expression of AR in prostate cancer cells [41]. Overexpression of AR in PC3 cells has been shown to influence cell invasion and growth in vitro [42]. Since we have established a direct functional link between cystatin C and Erk2 activity, and we have demonstrated that PCa patients with low cystatin C levels and concomitant high levels of AR showed a tendency towards a worse outcome as compared to patients with high cystatin C and high AR levels, we therefore wanted to assess the cellular and molecular association between cystatin C and AR. We employed PC3 cells that lack the functional AR to investigate a cooperative role between AR and cystatin C. PC3 cells were co-transfected with AR expressing vector along with cystatin C siRNA vector designated as “siCysC, AR” or with a control vector designated as “sictr, AR”. In a similar fashion, PC3 cells were also co-transfected with a CMV vector and cystatin C siRNA (designated as “siCysC, CMV”) or a CMV expressing vector and a control siRNA (“sictr, CMV”). The transfected PC3 cells were then subjected for invasion assay. Overexpression of AR with concomitant knockdown of cystatin C resulted in a significant increase in the rate of invasion of PC3 cells compared with the controls (**Figure 7A–B**). Thus, these results suggest that PC3 cells that lack the cystatin C but have high level of AR may be more invasive than the control cells with normal level of cystatin C and AR.

## Discussion

In the present study, we have shown that *in vitro* silencing of cystatin C by specific siRNA increased cancer cell invasion in cooperation with Erk2 and AR signalling. We unravelled novel molecular mechanisms by which cystatin C affects tumor cell invasion demonstrating that cystatin C expression was downregulated in primary prostate tumors compared with benign tissues in 448 patients. Using a TMA comprising benign and tumor samples from 448 patients, we showed an inverse correlation between cystatin C and MMP2 expression. Several studies have convincingly demonstrated that cystatin C is an important inhibitor of cathepsin B and tumor cell invasion [43], [44]. Cathepsin B influences tumor microenvironment by degradation of extracellular matrix and by activation of other proteolytic enzymes such as pro-urokinase-type plasminogen activator (pro-uPA) and matrix metalloproteases (MMPs) so that tumor cells can actively invade and metastasize[45]. Overexpression of cystatin C has been shown to inhibit the invasive potential of human melanoma and glioblastoma cell lines [36], [43]. Our present finding from the investigation of clinical materials indicates that there is a direct link between cystatin C and extracellular matrix protein MMP2 in prostate cancer. We have not investigated the role of cystatin C in modulation of cathepsin B activities in prostate cancer cells, however it is an open possibility considering the similar correlations in other type of tumors. Proteome signatures that are hallmarks of proteolysis revealed cleavage of many known MMP-2 substrates in the cellular context. Proteomic evidence of MMP-2 processing of novel substrates was found. Cystatin C protein is one of the substrate that is cleaved by MMP-2 [40]. We provide further evidence supporting a previously proposed role of cystatin C to prevent tumorigenesis and cancer cell invasiveness, possibly due to its ability to inhibit the activity of extracellular matrix proteins [46], [47].

Our functional analysis in PC-3 cells further demonstrated that inhibition of cystatin C via siRNA-mediated knockdown resulted in a significant increase in the rate of invasion of PC3 cells. This observation is consistent with previous demonstration in primary prostatic tumors where the most invasive tumors had very low or undetectable cystatin C expression. Thus, cystatin C may be functionally important for cells to maintain normal behaviour, which is in concordance with our present results that PC3 cells overexpressing cystatin C via transient transfection displayed less invasive phenotypes.

The Erk-MAPK pathway is a common signaling mechanism for multiple growth factors that are involved in metastatic spread and drug-resistance [48]. By employing siRNA against cystatin C, we showed that cystatin C was inversely associated with the invasive behavior of PC3 cells, and that cystatin C was involved in the regulation of Erk kinase activity. We revealed that the increased invasion rate of PC3 cells caused by silencing of cystatin C, was specifically blocked by inhibition of Erk2. Erk-MAPK pathway has been shown to be a target for anticancer drugs [49] and Erk2 effects are strongly interrelated with downstream target, Elk-1 [50], [51]. We observed a sustained cystatin C-dependent Erk activation in PC3 cells and it is unclear whether this is due to a convergence of AR and growth factor signalling pathways. We speculate that cystatin C may inhibit the protease-dependent release of growth factors or regulate activity status of receptor or phosphatases to modulate Erk2 activity. Here, we show that knockdown of Erk activity decreased the invasion rate of prostate cancer cells, which is consistent with the previous work in vitro and in vivo [52]. It has also been shown that inhibition of Erk1/2 suppressed *in vivo* invasiveness of a human squamous cell carcinoma cell line[53]. Moreover in pancreatic cancer cells TGFβ treatment caused an epithelial-mesenchymal transition that was associated with a more invasive phenotype and with the activation of ERK-signaling cascade[54]. In prostate cancer, constitutive activation of MAPK was related to the progression to androgen independence[55]. Our finding provides evidence that cystatin C regulates Erk activity in PC3 cells and that loss of cystatin C might lead to an uncontrolled increase in the activity of MAPK/Erk signaling cascades, and thereby result in an increase in invasion of PC3 cells.

Understanding multiple pathways that cooperatively contribute to tumor progression is of utmost importance for development of new therapeutic agents in prostate cancer. In the present study, we explored whether cystatin C is related to AR in androgen-independent tumor cell invasion and metastasis. Our findings suggest that loss of cystatin C and overexpression of AR may be two cooperative events in the progression of prostate cancer. This hypothesis was supported by our results from TMA studies of primary prostate cancer and benign prostatic tissue from 448 patients. We show that tumors with low levels of cystatin C and high AR levels exhibited a worse clinical outcome compared with patients with high levels of cystatin C and AR. Overexpression of AR is one of the mechanisms utilized by castration resistant prostate cancer cells, to overcome the growth inhibitory effects of hormone depletion therapy or other chemotherapy [56]. Here we show that decreased expression of cystatin C with concomitant overexpression of AR leads to further enhancement of cell invasion. It has been shown that inhibition of Erk1/2 activity in prostate cancer cell resulted in the reduction of AR gene expression [41]. Given that cystatin C mediates the activity of MAPK/Erk1/2, cystatin C may also contribute to the regulation of AR expression through MAPK/Erk1/2 signalling. In conclusion, our studies provide, new insights into the mechanisms of cystatin C actions in prostate cancer cells, and may provide valuable information on cancer targets for therapeutic interventions.

## Materials and Methods

### Tissue Specimens and Tissue Microarray

Benign and malignant tissue specimens from a consecutive series of 448 patients undergoing radical prostatectomy for localized prostate cancer at Malmö University Hospital, Sweden, between 1988 and 2003 were mounted in 17 tissue microarray blocks using a manual tissue arrayer (Beecher Inc, Wisconsin WI) as previously described[39]. One section form each block was stained with hematoxylin/eosin and examined by a National Board licensed Pathologist for the presence of tumor, including Gleason grading of each individual core. The study was approved by the Ethic's committee at Lund University (#LU 909/03) by December 17, 2003 and the Helsinki Declaration of Human Rights was strictly observed. Patients were informed retrospectively by advertisement in the local newspaper that their tissue samples were to be used for TMA studies. The TMA was constructed years after prostatectomy. In this study, the correlation between the expression of cystatin C and clinical parameters were investigated. The clinical parameters of the patients include: pretreatment of PSA level, Gleason score, clinical and pathological T stage, surgical margin status. Biochemical recurrence, was defined as a detectable level of PSA at >0.2 ng/ml as previously described [57], [58] and confirmed by a subsequent increasing value. Overall, cancer-specific, and recurrence-free survivals were assessed. Overall survival with follow up time ranging from 1 to 200 months were recorded.

### Cell Culture and Treatment

Human prostate cancer cell lines, LNCaP, PC-3 and DU-145 were purchased from the American Type Culture Collection (Manassas, VA, USA). Cells were grown in RPMI 1640 medium (LNCaP and DU-145) and F12-HAM's medium (PC-3) (PAA Laboratories GmbH, Linz, Austria) supplemented with 10% fetal calf serum (Sigma Chemical, St Louis, MO, USA), penicillin (100 U/mL) and streptomycin (100 µg/mL) (PAA Laboratories, Austria).

The Erk2 selective inhibitor, 3-(2-aminoethyl-5(4-ethoxyphenyl)methylene)-2,4-thiazolidinedione, HCl) (Calbiochem, Gibbstown, NJ) as previously described [59] was dissolved in DMSO and was used at concentration of 25 µM. Cells were pre-treated for 10 minutes with inhibitor and seeded for invasion assay at concentration of 2×10^5^ PC3 cells per well. The cell invasion was tested after 20 hours as described below.

### Transfections and siRNA Treatment

Cells were seeded at 40–50% confluence onto 6-well plates. siRNAs against cystatin C and Smad2 and negative controls were purchased from Ambion (Austin, TX). Five nM of siRNA was used for each transfection with Lipofectamine 2000 in Opti-MEM (Invitrogen, Carlsbad, CA). Cells were harvested and used for subsequent assays after 24 hours.

Vector with AR cDNA was kindly provided by Dr. Steven Balk (BIDMC, Harvard Medical School) and was previously described [60]. Empty pcDNA3.1 control plasmid was purchased from Invitrogen. Cells were transfected with Amaxa nuclofection Kit as previously described [39] and the transfection efficiency was 40–50% as measured with GFP control plasmid. In the co-transfection studies with siRNA and plasmids, transfections were carried out with nucleofection techniques as previously described[61].

Cystatin C cDNA was amplified from PC3 cells with the following primers:

F: 5′CTC G'AATTC c ctc tcg cct gcg ccc cac tcc; and R: 5′GC'GGCCGC gg cacaggccag cccggtac and subcloned into pcDNA3.1 vector between EcoRI and NotI restriction sites. The subcloning was verified by sequencing and restriction sites analysis. pcDNA3.1 control and cystatinC-pcDNA3.1 vectors were used to establish the stable PC3 cell line. Cells were transfected with Lipofectamine 200 as described above and selected on 200 µg/ml neomycin for 2 weeks. The expression of cystatin C in the clones of PC3 cells was verified by immunoblotting.

### Immunoblotting

Cells were lysed in ice-cold lysis buffer (150 mM NaCl, 50 mM Tris-HCl pH 7.5, 1% NP-40, 10 mM NaF, 1%SDS, 1 mM EDTA pH 8.0, 10 mM phenylmethylsulfonyl fluoride (PMSF) and the protease inhibitor cocktail Complete Mini (Roche, Basel, Switzerland). Samples were centrifuged for 20 min, 14000×g at 4°C and clear supernatants were collected. 20 µg of each protein samples were separated on 12% SDS-PAGE gel followed by transfer to nitrocellulose membrane Hybond^TM^ECL^TM^ (Amersham Pharmacia Biotech, Buckinghamshire, U.K.). The membranes were probed with appropriate primary antibodies followed by horseradish peroxidase (HRP)-conjugated secondary antibodies (Amersham Life Science, Alesbury, U.K.) at the dilution 1∶5000 and visualized using the Enhanced ChemiLuminescence detection system (ECL Plus) and ECL films (Amersham Pharmacia Biotech).

### Antibodies

The following antibodies were used: polyclonal rabbit antibodies against cystatin C (code no. A0451) and non-immune rabbit IgG (code no. X0936) were purchased from DAKO A/S (Glostrup, Denmark); monoclonal mouse anti-MMP2 (Chemicon, Millipore, CA), monoclonal anti-MMP2 (LabVision, NeoMArkers, Clone VC2, CA), polyclonal rabbit anti-phopho-Erk1/2, anti-phospho-Smad2 (Ser465/467), polyclonal anti-Erk1/2 (Cell Signaling Technology, MA), monoclonal goat anti-beta actin, mouse monoclonal anti-phospho-(Ser 383)-Elk1 (Santa Cruz Biotechnology), biotinylated secondary antibodies, anti-rabbit and anti-mouse IgGs were included in DAKO ChemMate kit (code no. K5003, BioTek™Solutions, Winooski, VT). Anti-mouse, anti-goat, anti-rabbit HRP conjugated were from Amersham Life Science (Alesbury, U.K.). A polyclonal rabbit antiserum raised against human cystatin C, purified from urine (code 8206) [62] was used as capture antibody in ELISA.

### Immunohistochemistry

Immunohistochemistry on tumor tissue microarrays as wells as evaluation and scoring was performed as previously described[61]. The specimens were viewed with an Olympus BX51 microscope at magnification of 40×. Each core of TMA was scored for the intensity of staining in the scale of 0–3 (0-negative, 1- moderate, 2- strong, 3- very strong). Positive staining in at least 60% of tumor or benign areas was considered to be significant to score to any of the group. For evaluation of survival curves, all the samples were divided to two groups: for the low (intensity score 0–1.5) and high (intensity score 2–3) cystatin C expression.

### Invasion Assay

The invasion of PC3 cells was measured by using the Transwell chambers (Chemicon, Millipore, CA) according to the manufacturer's protocol. Briefly, the cells were transfected with siRNA and/or plasmids and after 24 hours were seeded onto the membrane of the upper chamber of the transwell at a concentration of 2×10^5^/ml in 500 µl of HAM's F-12 medium. The medium in the upper chamber was serum-free. The medium at the lower chamber contained 10% Foetal Calf serum as a source of chemoattractants. Cells that passed through the Matrigel coated membrane were stained with Cell Stain Solution containing crystal violet supplied in the Transwell Invasion assay (Chemicon, Millipore, CA) and photographed after 20 hours of incubation. Absorbance was measured at 562 nm by ELISA reader after dissolving of stained cells in 10% acetic acid.

### Cystatin C ELISA

Quantitation of cystatin C in the supernatants of prostate cancer cell lines by a specific ELISA was performed as we have previously described[63].

### Statistical Analysis

T-student Test for assessing the significance of in vitro experimentation; Wilcoxon's rank sum test was used to assess the differences in the expression of cystatin C between benign and malignant specimens; Kaplan Meier survival analysis were performed. The two-sided log-rank test was used to test association between variables and clinical and biochemical recurrence, with 95% confidence intervals. Spearman rank correlation test was used to evaluate possible pair-wise correlations between the expression of cystatin C and MMP2. The Statistical Package for the Social Sciences (SPSS) version 11.5 (SPSS Inc. Chicago, IL) was used for the analysis. P-values<0.05 were considered as statistically significant.
